# RNA-Seq Implies Divergent Regulation Patterns of LincRNA on Spermatogenesis and Testis Growth in Goats

**DOI:** 10.3390/ani11030625

**Published:** 2021-02-26

**Authors:** Dongdong Bo, Xunping Jiang, Guiqiong Liu, Ruixue Hu, Yuqing Chong

**Affiliations:** 1 Laboratory of Small Ruminant Genetics, Breeding and Reproduction, College of Animal Science and Technology, Huazhong Agricultural University, Wuhan 430070, China; Dongdbo@foxmail.com (D.B.); xpjiang@mail.hzau.edu.cn (X.J.); rxhu@webmail.hzau.edu.cn (R.H.); YQChong@webmail.hzau.edu.cn (Y.C.); 2 Key Laboratory of Agricultural Animal Genetics, Breeding and Reproduction, Ministry of Education, Wuhan 430070, China

**Keywords:** long intergenic non-coding RNAs, testicular development, phase transition, growth, spermatogenesis, goat

## Abstract

**Simple Summary:**

Long intergenic non-coding RNAs (lincRNAs) can regulate testicular development by acting on protein-coding genes. Therefore, it is important to explore the expression patterns and roles of lincRNAs during the postnatal development of the goat testis. In this study, the testes of Yiling goats with average ages of 0, 30, 60, 90, 120, 150, and 180 days postnatal were used for RNA-seq. In total, 20,269 lincRNAs were identified, including 16,931 novel lincRNAs. Using weighted gene co-expression network analysis, seven time-specifically diverse lincRNA modules and six mRNA modules were identified. Dramatically, the down-regulation of growth-related lincRNAs was nearly one month earlier than the up-regulation of spermatogenesis-related lincRNAs, while the down-regulation of growth-related protein-coding genes and the correspondent up-regulation of spermatogenesis-related protein-coding genes occurred at the same age. Moreover, potential lincRNA target genes were predicted. Moreover, key lincRNAs in the process of testis development were predicted, such as ENSCHIT00000000777, ENSCHIT00000002069, and ENSCHIT00000005076. In the present study, the divergent regulation patterns of lincRNA on spermatogenesis and testis growth were discovered. This study can improve our understanding of the functions of lincRNAs in the regulation of testis development.

**Abstract:**

Long intergenic non-coding RNAs (lincRNAs) regulate testicular development by acting on protein-coding genes. However, little is known about whether lincRNAs and protein-coding genes exhibit the same expression pattern in the same phase of postnatal testicular development in goats. Therefore, this study aimed to demonstrate the expression patterns and roles of lincRNAs during the postnatal development of the goat testis. Herein, the testes of Yiling goats with average ages of 0, 30, 60, 90, 120, 150, and 180 days postnatal (DP) were used for RNA-seq. In total, 20,269 lincRNAs were identified, including 16,931 novel lincRNAs. We identified seven time-specifically diverse lincRNA modules and six mRNA modules by weighted gene co-expression network analysis (WGCNA). Interestingly, the down-regulation of growth-related lincRNAs was nearly one month earlier than the up-regulation of spermatogenesis-related lincRNAs, while the down-regulation of growth-related protein-coding genes and the correspondent up-regulation of spermatogenesis-related protein-coding genes occurred at the same age. Then, potential lincRNA target genes were predicted. Moreover, the co-expression network of lincRNAs demonstrated that ENSCHIT00000000777, ENSCHIT00000002069, and ENSCHIT00000005076 were the key lincRNAs in the process of testis development. Our study discovered the divergent regulation patterns of lincRNA on spermatogenesis and testis growth, providing a fresh insight into age-biased changes in lincRNA expression in the goat testis.

## 1. Introduction

In the mammalian genome, only about 2% of DNA sequences are protein-coding genes, the rest are non-coding regions, including long non-coding RNAs (lncRNAs) [1]. LncRNAs are defined as a series of long-chain RNAs without protein-coding potential [2,3]. The feature being longer than 200 nt distinguishes lncRNAs from other types of non-coding RNA, such as microRNA (miRNA), piwi-interacting RNA (piRNA), transfer RNA (tRNA), and small nucleolar RNA (snoRNA). In addition, long-chain non-coding RNAs such as ribosomal RNAs (rRNAs) and nuclear RNAs are not included in lncRNAs [4,5].

Despite the lower expression level and coding potential of lncRNAs compared with protein-coding genes, lncRNAs have been proved, by increasing amounts of evidence, to be critical regulators of important life activities. For example, the famous H19, as an imprinted maternally expressed lncRNA, is widely involved in the growth and reproduction processes [6,7]. Another well-documented lncRNA, Xist (X inactive-specific transcript), also regulates the dose compensation effect and X chromosome inactivation [8]. With the extensive implementation of lncRNA research in the past two decades, lncRNAs have been found to function in embryonic development [9], muscle growth [10], immunity [11], metabolism [12], and gametogenesis [13].

According to their locational relationship with known coding genes, lncRNAs are divided into genic lncRNAs and intergenic lncRNAs (long intergenic non-coding RNAs, lincRNAs). In addition to the common properties shared by other members of lncRNA family, lincRNA are also non-overlapping with the annotated coding genes [14]. LincRNAs were mined from unknown regions of the genome, which is significant for the evolving understanding of the functions of the non-coding RNA world.

Recently, emerging evidence revealed the deep involvement of lincRNAs in testicular development. The abnormal expression of lincRNAs leads to development disorders of human reproductive system, and cause some serious diseases, such as Klinefelter’s syndrome [15], cryptorchidism [16], and Kallmann syndrome [17]. In addition, the roles of lincRNAs in testicular development have been repeatedly mentioned—they are widely involved in sex determination [18], embryonic testicular development [19], postnatal testicular growth [20], and spermatogenesis [21].

Our previous study has shown that the process of testicular development can be divided into the organ growth phase and the spermatogenesis phase. The expression of genes related to organ growth decreased, while at the same time, genes related to spermatogenesis were up-regulated and maintained high expression levels [22]. As a regulator of mRNA expression, lincRNAs usually exhibit a similar expression pattern to mRNAs. However, the expression patterns of lincRNAs in the goat testis remained largely unexplored. Therefore, this study aims to demonstrate the expression patterns and roles of lincRNAs during the postnatal development of the goat testis, hoping to provide new insights and valuable information for further investigation of lincRNA function in testis development and spermatogenesis.

## 2. Materials and Methods

### 2.1. Ethics Statement

This study was conducted according to the guidelines of The Scientific Ethic Committee of Huazhong Agricultural University (HZAUGO-2017-006).

### 2.2. Sampling

For this study, 21 male Yiling goats with average ages of 0, 30, 60, 90, 120, 150, and 180 days postnatal were randomly selected and purchased from the Changyang Yongxing Ecological Husbandry Co., Ltd. (Yichang, Hubei, China). Each age group comprised three goats. The goats were raised in the same environmental conditions. The goats were sedated intramuscularly by using 0.1 mL/kg xylazine hydrochloride (Shengda, Changchun, Jilin, China), and the testicles were surgically collected, frozen immediately in liquid nitrogen, and then stored at −80 °C. The details of experimental goats and sample collection methods were described in our previous study [22].

### 2.3. RNA Isolation, Library Construction, and Sequencing

Total RNA was extracted from testicular tissue. Detailed transcriptome library construction and sequencing methods were described previously by Bo et al. [22]. The raw data of transcriptome sequence have been deposited in the Genome Sequence Archive [23] in BIG Data Center [24], Beijing Institute of Genomics (BIG), Chinese Academy of Sciences (accession code: CRA002191) at https://bigd.big.ac.cn/gsa, accessed on 20 December 2019. The quality of raw sequencing reads was determined using the FastQC software [25] and adapters were trimmed by the Trimmomatic [26].

### 2.4. Mapping and Assembling

Clean pair-end reads of high quality were mapped to the goat reference genome (ftp://ftp.ensembl.org/pub/release-93/fasta/capra_hircus/dna/Capra_hircus.ARS1.dna.toplevel.fa.gz, accessed on 8 March 2019) using the TopHat software [27] with default parameters. The annotation for genes were downloaded from the Ensembl database (ftp://ftp.ensembl.org/pub/release-93/gtf/capra_hircus/Capra_hircus.ARS1.93.gtf.gz, accessed on 8 March 2019). The Cufflinks program [27] was used to assemble the transcripts. 

### 2.5. Coding Potential Analysis of Transcripts

We used three analytic tools to screen candidate lincRNAs, including Coding-Non-Coding Index (CNCI) [28], Coding Potential Calculator (CPC) [29], and PfamScan [30]. We used the following steps to identify lincRNAs from the goat testicular transcriptome: (1) only transcripts with ‘u’ category categorized by Cuffmerge [27] which indicated intergenic transcripts were retained; (2) transcripts with a single exon or those less than 200 nt in length were filtered; (3) only transcripts with CPC value < 0 in both strands were retained; (4) only transcripts without coding potential detected by CNCI are retained; and (5) transcripts that contained known protein domain were filtered. To accomplish this, we translated transcript sequences into six possible protein sequence with the ‘Transeq’ tool in European Molecular Biology Open Software Suite (EMBOSS) [31], and then transcripts with any possible protein sequence significantly (E-value < 1 × 10^−5^) hit in the Pfam database [32] were filtered. Transcripts that had detectable expression in all three datasets were selected to minimize false positive.

### 2.6. Principal Component Analysis (PCA) and Correlation Analysis of Samples

The abundance of each gene was defined using the fragments per kilobase of exon per million fragments mapped (FPKM), and the FPKM matrix of all the lincRNAs was inputted for the analyses of this section. Before analysis, lincRNAs with a sum of FPKM values less than 1 were removed. PCA and correlation analysis were performed with R [33], and the results were visualized using the R packages ‘ggplot2′ [34] and ‘pheatmap’ (https://cran.r-project.org/web/packages/pheatmap, accessed on 9 May 2019), respectively. 

### 2.7. Differential Expression Analysis

Read count matrices was exported using the HTSeq-count program [35]. Then, we used the R package ‘DESeq2′ [36] to conduct differential expression tests on lincRNAs. Transcripts with *q* ≤ 0.05 and |log_2_(fold change)| ≥ 1 were assigned as significantly differentially expressed lincRNAs (DELs) between two age groups. The differentially expressed protein-coding genes (DEPGs) were obtained from the results of our previous study [22]. We then combined the DELs and DEPGs in different groups to one DEL and DEPG union set, respectively, and did the following analysis.

### 2.8. Quantitative Real-Time PCR (qRT-PCR) Validation

The total RNA samples were reverse-transcribed using the PrimeScript RT reagent Kit with gDNA Eraser (Toyobo, Osaka, Japan). The reaction system contained 1 μg of RNA, 2 μL of 5 × RT buffer, 0.5 μL of primer mix, 0.5 μL of enzyme mix and deionized water in a final volume of 10 μL. The reaction was carried out at 37 °C for 15 min and 98 °C for 5 min. The cDNA was diluted five times and then used as the template of qRT-PCR. The specific primers for qRT-PCR were designed using NCBI Primer-BLAST [37]. The qRT-PCR reaction system contained 5 μL of 2 × SYBR Green qPCR Mix (Aidlab Biotechnologies, Beijing, China), 1 μL of cDNA, 0.5 μL of upstream primer, 0.5 μL of downstream primer, and deionized water in a final volume of 10 μL, and the experiment was conducted using a CFX384 Touch Real-Time PCR Detection System (Bio-Rad, Hercules, CA, USA). Meanwhile, the PCR was conducted at 95 °C for 5 min, followed by 40 cycles of 95 °C for 10 s, 60 °C for 20 s, and 72 °C for 20 s. The gene expression level was determined by the 2^−ΔΔCt^ algorithm, and the goat beta-actin (*ACTB*) gene was used as an internal control [38]. Each sample had three biological replicates, and the gene expression level was presented as the means ± standard errors (*SE*) (*n* = 3). The primer sequences of the selected genes are shown in Table 1. The visualization of qRT-PCR was performed using the software GraphPad prism [39].

Correlation analysis of RNA-Seq and qRT-PCR results was carried out using R [33]. Comparison of changes in lincRNA expression (represented as log2) derived from RNA-Seq and qRT-PCR revealed a significant correlation (Pearson’s *p* = 0.016 at the *α* level of 0.05) and visualized using the R package ‘ggpubr’ [40].

### 2.9. WGCNA Analysis

Gene co-expression networks were constructed using the R package ‘WGCNA’ (v1.67) [41]. The FPKM values of the DEL and DEPG union set were inputted for the analysis. The lincRNA modules (LMs) and mRNA modules (MMs) were clustered using the automatic network construction function, ‘blockwiseModules’, with default settings. The eigen lincRNAs were chosen for each LM to represent the expression pattern. *Ki*, which means intramodular connectivity, was calculated from the sum of its connection strengths with all the other lincRNAs in the same module, used for identifying hub lincRNAs of each module. Intramodular hub lincRNAs were selected based on a high connectivity (*Ki* > 0.9). The co-expression networks of hub lincRNAs were produced using ‘igraph’ package (https://igraph.org/r/, accessed on 23 December 2019) on the R platform [33].

### 2.10. Functional Enrichment Analysis

The FPKMs of lincRNAs and mRNAs were calculated for Pearson’s correlation coefficients (PCC) using R [33]. Highly correlated mRNA pairs were screened with a threshold that the absolute value of *r* no less than 0.8 and the *p* value less than 0.05. The highly correlated mRNAs were used for gene ontology (GO) enrichment and the target gene prediction of lincRNAs. Because of the poor annotations of goat genome, the goat gene IDs were converted into cow orthologues using the Ensembl BioMart website [42] and the cow orthologues of “one2one” homology type were uploaded to the PANTHER online tool [43,44] for GO enrichment analysis.

### 2.11. The Prediction of the Target Genes of LincRNAs

The regulatory relationship between lincRNAs and mRNAs was predicted using regression analysis. The regression model is shown as

*M* = *A*_1_ × *L*_1_ + *A*_2_ × *L*_2_ + … + *A_n_* × *Ln* (1)

where *M* represents the FPKM vectors of mRNAs; *L*_1_, *L*_2_, …, *L_n_* represent the FPKM vectors of lincRNAs that highly correlates with the same mRNA; *A*_1_, *A*_2_, …, *A_n_* represent the regression coefficient of *L*_1_, *L*_2_, …, *L_n_*. The circular presentation of the association between lincRNA and mRNA modules was visualized using the Circos website [45].

## 3. Results

### 3.1. Identification of LincRNAs in the Goat Testis

**Figure 1:**
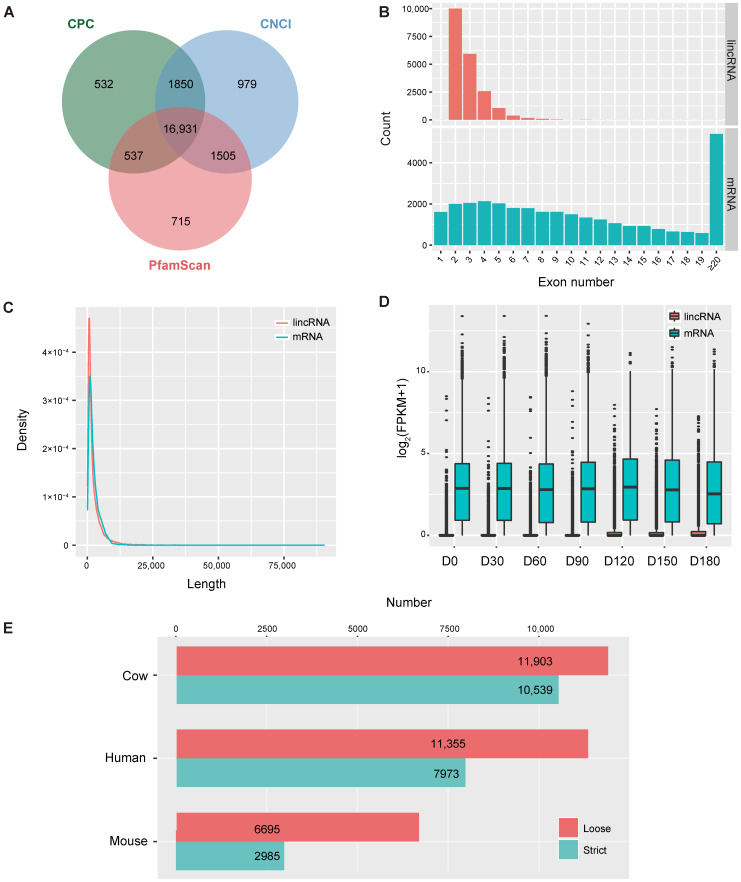
Identification of long intergenic non-coding RNAs (lincRNAs). (**A**) Identification of the coding potential of transcripts using CNCI, CPC and PfamScan. (**B**) Compared with mRNAs, lincRNAs had fewer exons. (**C**) Compared with the mRNAs, the lincRNAs were shorter. (**D**) Transcriptional levels of lincRNAs and mRNAs. The transcription levels of lincRNAs and mRNAs at each age are represented by the mean value of the three individuals. (**E**) Number of goat testicular lincRNAs that are homologous to NONCODE lincRNAs in the cow, human, and mouse with a loose (E-value < 1 × 10^−3^) and strict (E-value < 1 × 10^−10^) threshold by BLASTN.

After alignment, assembly, and transcript screening, we used the CNCI, CPC, and PfamScan to predict the coding potential of transcripts and screened 16,931 novel goat testicular lincRNAs (Figure 1A and Supplementary Table S1). In addition, the expressions of 3338 goat lincRNAs archived on the Ensembl database were detected in this study. Therefore, a total of 20,269 goat testicular lincRNAs were found. The lincRNAs contained fewer exons than mRNA (Figure 1B and Supplementary Table S2). Moreover, the lincRNAs in our dataset were shorter than mRNAs (Figure 1C and Supplementary Table S2).

The expression of lincRNAs remained at low levels from 0 DP to 90 DP but remarkably increased at 120 DP and the overall transcription level of lincRNAs remained after 120 DP (Figure 1D and Supplementary Table S2). Moreover, a lower expression of lincRNAs was observed at each age compared with mRNAs (Figure 1D and Supplementary Table S2).

To further explore the conservation of goat testicular lincRNAs, we downloaded 337,593, 4,016,128, and 5,918,797 lncRNAs specific for cow, mouse, and human, respectively, from the NONCODE database [46], followed by an alignment analysis. Among them, 11,903, 11,355, and 6695 goat testicular lincRNAs were aligned to 4145 cow, 46,870 human, 18,692 mouse lncRNAs, respectively (Figure 1E). Note that the homologs identified between goat testicular lincRNAs and the NONCODE cow lncRNAs were not significantly reduced by increasing the BLAST [47] stringency, while those of the human and mouse lncRNAs were significantly decreased (Figure 1E).

### 3.2. The Expression Profile of LincRNAs

**Figure 2:**
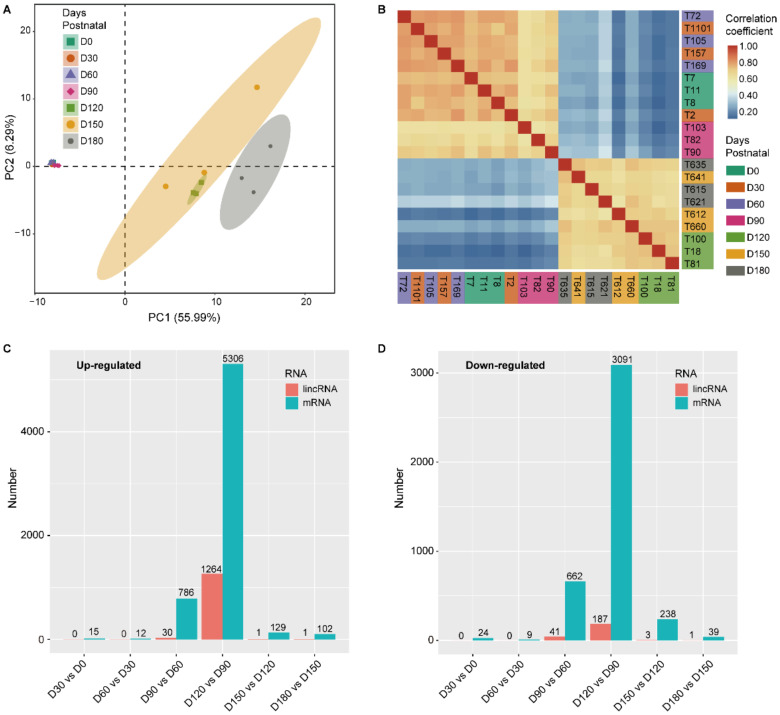
The discrete expression patterns of lincRNAs and mRNAs. (**A**) Principal component analysis (PCA) of 21-pair distinct samples across the seven ages based on normalized lincRNA expression levels. The samples were clustered by age. (**B**) Heatmap of correlation coefficient for 21 samples based on the lincRNA expression level. The samples were grouped by hierarchical clustering and the dendrogram was not shown. The color of the blocks below the sample names represents the age group to which each of the samples belonged. (**C**,**D**) Number of differentially expressed lincRNAs and mRNAs between neighboring age groups.

The PCA demonstrated that the samples obtained at 0, 30, 60, and 90 DP formed a distinctive cluster, and the samples obtained at 120, 150, and 180 DP formed another cluster (Figure 2A and Supplementary Table S3). This result was confirmed by the hierarchical clustering (see the hierarchical clustering heatmap in Figure S1) and the correlation analysis (Figure 2B and Supplementary Table S3). In contrast, the samples were clustered into three classes (0–60 DP, 90 DP, and 120–180 DP classes) in our previous study [22] based on the transcriptional level of protein-coding genes.

The DELs between any two adjacent age groups (0, 30, 60, 90, 120, 150, and 180 DP) were identified as having *q* < 0.05 and |log_2_(fold change)| ≥ 1 (Figure 2C,D and Supplementary Table S4). The expression of lincRNAs changed the most in the 90–120 DP stage (1451 DELs), followed by the 60–90 stage (71 DELs). The ratio between the DEL numbers in the periods of 90–120 and 60–90 DP was 20.44 (Figure 2C,D), approximately 4-fold that of DEPG, which was only 5.80 [22]. The above results suggest that 90–120 DP may be the first major shift in lincRNA expression, later than that of protein-coding genes (60–90 DP).

Moreover, the 90–120 DP stage was observed with much more up-regulated lincRNAs (UL) than down-regulated lincRNAs (DL) (1264 up-regulated and 187 down-regulated lincRNAs), while the number of up-regulated and down-regulated protein-coding genes was similar at this stage [22]. Interestingly, the majority (99.21%) of ULs at the 90–120 DP stage were non-differentially expressed in the previous stages (0–30 DP, 30–60 DP, and 60–90 DP).

**Figure 3:**
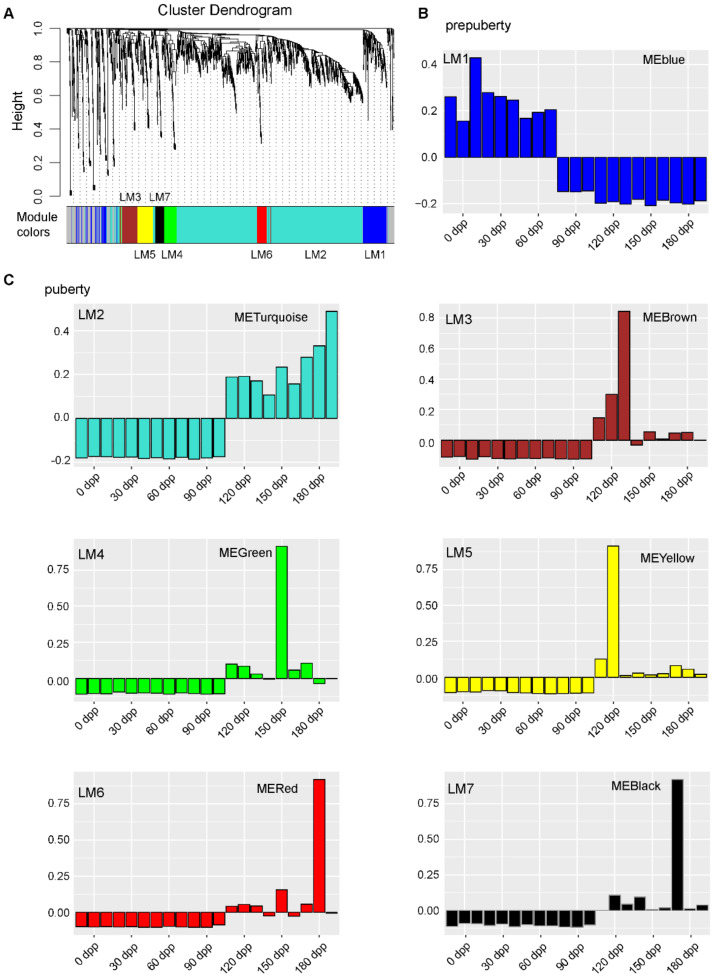
LincRNAs are clustered into temporal-specific modules by the weighted gene co-expression network analysis. (**A**) Hierarchical cluster dendrogram of all lincRNAs modules. Modules corresponding to branches are labeled with colors indicated by the color bands underneath the tree. (**B**,**C**) Eigengene bar plot of lincRNA modules. Samples were sorted by days postnatal in the order of 0, 30, 60, 90, 120, 150, and 180 DP. LM represents lincRNA module.

To characterize the dynamic changes of differentially expressed lincRNA and mRNA expression, we clustered all their expression patterns (1656 lincRNAs and 8144 mRNAs) by the WGCNA method [41]. We identified seven main lincRNA transcriptional modules, each represented by a characteristic expression pattern (Figure 3A and Supplementary Table S5). On the other hand, six main mRNA transcriptional modules were also identified (Supplementary Figure S2 and Table S6). Each lincRNA and mRNA module was explored by eigengene value graphing described by the color corresponding to the cluster dendrogram (Figure 3B,C; Supplementary Table S5; Figure S2; and Table S6).

According to their temporal expression patterns, the lincRNA modules were separated into two classes—prepuberty and puberty modules. Prepuberty modules were characterized by a remarkably higher expression in 0–60 DP and lower expression level in the subsequent periods (Figure 3B). The puberty modules demonstrated dramatically increased transcription levels at 120 DP, and maintained a high expression level in the subsequent periods (Figure 3C). In contrast to mRNA modules, more lincRNA modules were found, which indicates the diverse expression patterns and complex regulation of lincRNAs.

Among the lincRNA modules, the second largest lincRNA module, LM1 contains 167 lincRNAs. In this study, LM1 was the only prepuberty module (Figure 3B). The expression of lincRNAs in LM1 maintained a high level at 0, 30, and 60 DP, while their transcription levels dropped abruptly at 90 DP, and remained low at 90, 120, and 150 DP. All lincRNA modules except LM1 were puberty modules. This class of lincRNAs maintained a low transcriptional level from 0 to 90 DP, and a significant increment occurred at 120 DP. The highest transcription level of lincRNA in LM3 and LM5 appeared at 120 DP, while LM4 appeared at 150 days of age, and LM6 and LM7 appeared at 180 days of age. For the largest lincRNA module, LM2, the expression level gradually increased during the stage of 120–180 DP. This class of lincRNAs were likely to be widely involved in a series of life activities after the initiation of puberty. The lincRNAs in the puberty modules accounted for 87.20% of all DELs, indicating the high dynamics of testicular lincRNA expression at 120 DP.

For the mRNA modules, MM2 contained the most mRNAs (3716 mRNAs), followed by MM1 (3357 mRNAs). In contrast to the lincRNA modules, the expression patterns of the mRNA modules were more diverse (Supplementary Figure S2), which may indicate a more strictly regulated expression and more specific function of lincRNAs. The mRNA expression patterns of the MM1 and MM2 modules both changed dramatically at 120 DP. The MM1 module maintained a low expression level in the period of 0–90 DP, and turned to a continuous high expression level from 120 DP to 180 DP, while the expression pattern of the MM2 module was completely opposite (Figure S2).

### 3.3. The Time-Specific Expression of LincRNAs Contributed to the Transition of Testicular Development Stages

The correlation analysis between the modules and the traits was implemented to identify the key modules (Figure S3). As shown in Figure S3A, the LM1 and LM2 modules showed significant correlations with the testicular weight (*r* = −0.78 and *p* = 4 × 10^−5^ for LM1 and *r* = 0.94 and *p* = 3 × 10^−10^ for LM2).

The PCC between 20,269 lincRNAs and 27,271 mRNAs was computed based on their FPKM values in the 21 transcriptomes to explore the functions of testicular lincRNAs. A total of 3,041,298 co-expression pairs were detected between 4638 lincRNAs and 19,316 mRNAs at a stringency of *p* ≤ 0.01 and PCC ≥ 0.8. Enriched GO terms and pathways were further obtained for all mRNAs interacted with each lincRNA module.

**Figure 4:**
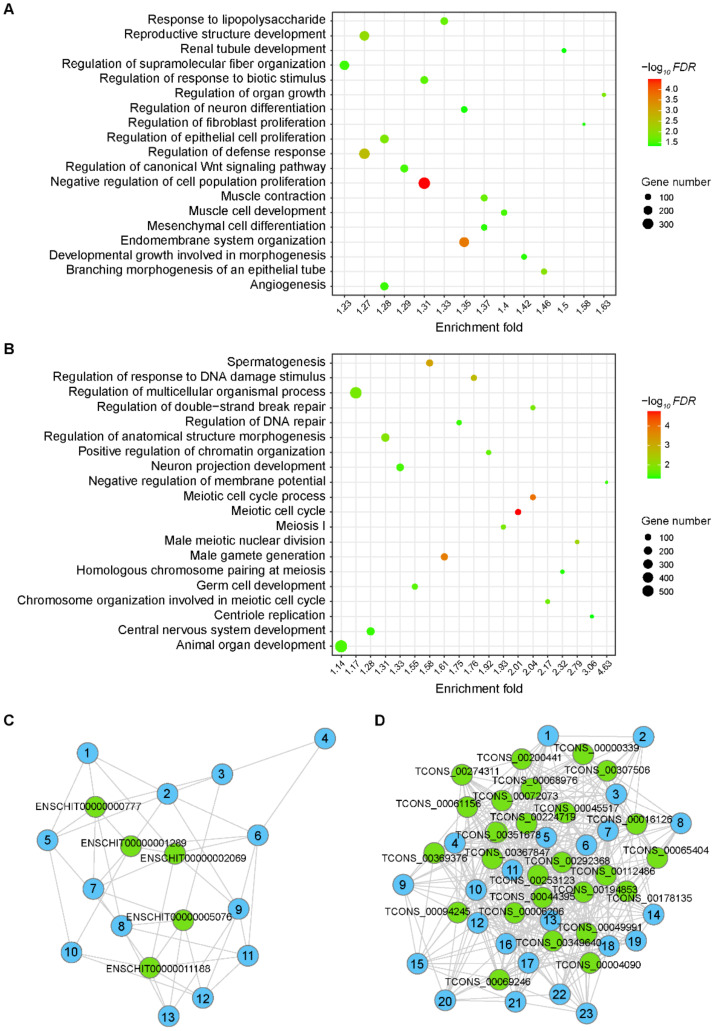
The function of lincRNA in key modules associated with testicular development. (**A**,**B**) Bubble chart presentation of the gene ontology (GO) terms for lincRNAs in modules LM1 (**A**) and LM2 (**B**). The function of each lincRNA was annotated by coexpressed mRNAs. The color degree of each dot represents the statistical significance of pathways (−log_10_*FDR*). The size of the dot represents the number of mRNAs. (**C**,**D**) The R package ‘igraph’ generated network-based visualization demonstrates the hub lincRNAs of module LM1 (A) and LM2 (B). The green dots represent the hub lincRNAs. The blue dots represent lincRNAs whose *Ki* value were slightly lower than hub lincRNAs and details were shown in Table S9.

For the prepuberty modules, the GO term “negative regulation of membrane potential” was the most enriched for LM1 lincRNAs (Supplementary Table S7). The lincRNAs in LM1 were mainly overrepresented in the organ growth, including the regulation of organ growth (“regulation of organ growth”, “developmental growth involved in morphogenesis” “embryonic organ morphogenesis”, and “regulation of cellular component size”), neurogenesis (“central nervous system neuron development”, “regulation of neuron differentiation”, and “modulation of chemical synaptic transmission”), tubular tissue growth (“branching morphogenesis of an epithelial tube” and “renal tubule development”), epithelial morphogenesis (“regulation of morphogenesis of an epithelium”, “regulation of epithelial cell proliferation”, and “cell junction organization”), connective tissue growth (“regulation of fibroblast proliferation”, “mesenchymal cell differentiation”, and “regulation of supramolecular fiber organization”), microtubule formation center (“microtubule organizing center organization”), protein metabolism (“positive regulation of ubiquitin-dependent protein catabolic process”), cell division (“negative regulation of mitotic cell cycle phase transition”, “mitotic cell cycle process” and “organelle assembly”), and muscle tissue development (“striated muscle cell differentiation”, “muscle cell development”, and “muscle organ development”) (Figure 4A and Supplementary Table S7). Terms related to cell apoptosis, lipid breakdown, immunity, angiogenesis, and DNA damage repair were also enriched for the lincRNAs in LM1 (Figure 4A and Supplementary Table S7).

The largest puberty module, lincRNAs in LM2 were mainly overrepresented in spermatogenesis (“male meiotic nuclear division”, “homologous chromosome pairing at meiosis”, “spermatogenesis”, and “germ cell development”), cilia growth (“centriole replication”), and DNA damage repair (“regulation of double-strand break repair” and “DNA repair”) (Figure 4B and Supplementary Table S8). Terms associated with protein quality control, metabolism, cell division, and apoptosis were also overrepresented for lincRNAs in LM2 (Figure 4B and Table S8).

Hub lincRNAs usually show high within-module connectivity. As shown in Figure 4C,D, five and twenty-six hub lincRNAs were detected in LM1 and LM2, respectively, using *Ki* > 0.8 as the cutoff (Supplementary Table S9).

For the mRNA modules, the GO term “piRNA metabolic process” (GO: 0034587, enrichment fold = 7, *FDR* = 2.6 × 10^−4^) was the most enriched for MM1 lincRNAs (Supplementary Figure S4). The mRNAs in MM1 were majorly overrepresented in the spermatogenesis, including meiosis (“meiotic nuclear division”), spermatid development (“DNA methylation involved in gamete generation” and “spermatid development”), sperm tail growth (“axonemal dynein complex assembly” and “flagellated sperm motility”), and fertilization (“single fertilization”). For the mRNAs in MM2, the GO term “cellular response to interleukin-15” (GO: 0071350, enrichment fold = 6.21, *FDR* = 3.17 × 10^−2^) was the most enriched (Supplementary Figure S4). The mRNAs in MM2 were mainly enriched in the GO terms associated with organ growth (Supplementary Figure S4).

### 3.4. Prediction of LincRNA Target Genes

In order to predict the potential regulatory effects of lincRNAs on protein-coding genes, regression analysis was conducted on the highly correlated lincRNA–mRNA pairs. After the stringent filtering, 89,199 lincRNA–mRNA pairs remained, containing 2645 lincRNAs and 15,256 mRNAs (Table S10). The network of lincRNA–mRNA pairs contained 52.80% of positive lincRNA–mRNA pairs and 47.20% of the negative pairs (Supplementary Table S10).

**Figure 5:**
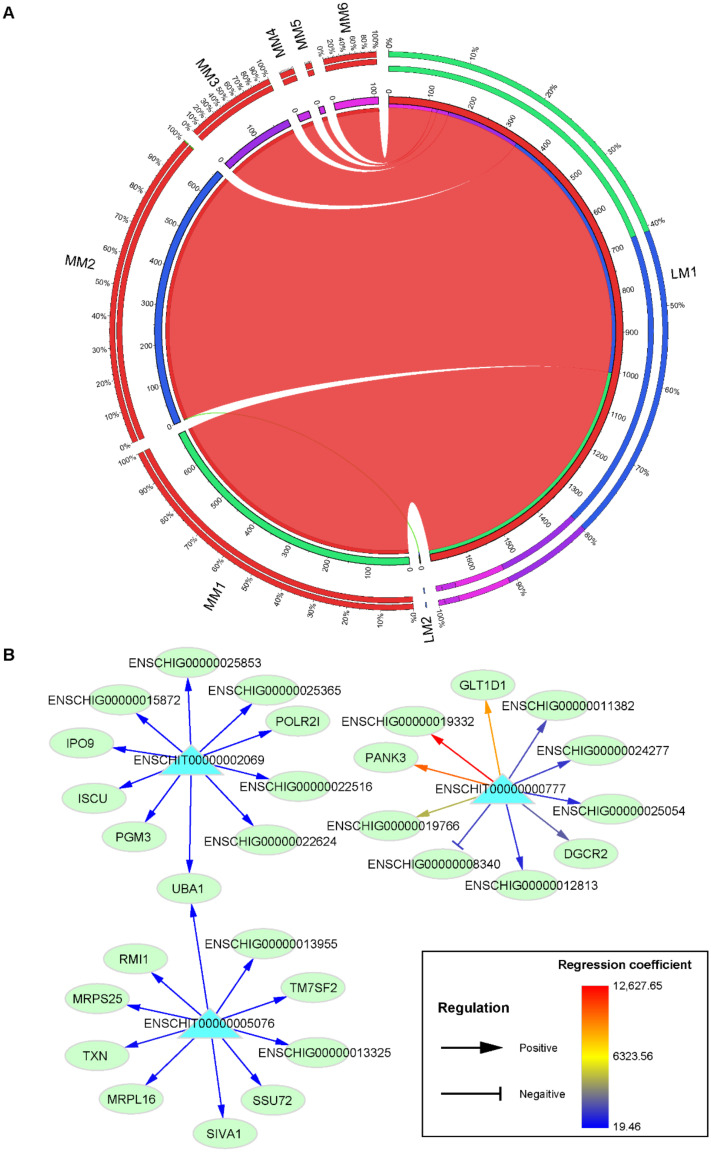
The prediction of the regulatory relationship between lincRNAs and mRNAs. (**A**) Circular presentation of the positive association between lincRNA and mRNA modules. The length of the inside cambered bar represents the number of lincRNAs and mRNAs. The outer cambered bar represents the proportion of lincRNAs and mRNAs with potential regulatory relationships to the number of total lincRNAs (LM modules) and mRNAs (MM modules) in the module to which they belonged. (**B**) The predicted regulatory relationship between hub lincRNAs of module LM1 (blue triangles) and mRNAs (green ellipses). The absolute regression coefficients were ranked and the top ten mRNAs with higher regression coefficients were selected for illustration. The arrow indicates the positive regulatory relationships and the T-shaped head indicates the negative regulatory relationships.

Next, we analyzed the module–module interaction between lincRNAs and mRNAs (Figure 5A and Supplementary Figure S5). Figure 5 shows the presence of a large number of LM1 lincRNAs that controlled mRNAs belonging to the module MM1 (high expression from 120 DP) and the module MM2 (low expression from 120 DP). In both positive and negative regulatory networks, module LM1 contained the most regulatory lincRNAs and mRNAs of module MM1 were most extensively regulated by lincRNAs (Figure 5A and Supplementary Figure S5).

### 3.5. qRT-PCR Test

**Figure 6:**
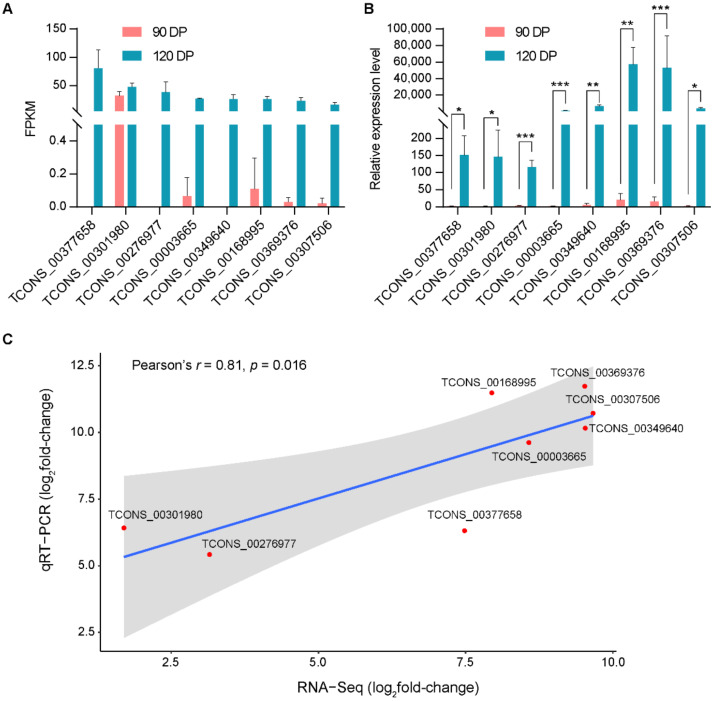
Validation of microarray results by qPCR analysis. (**A**) The results of RNA-seq of differentially expressed lincRNAs (DELs) of 120 DP vs. 90 DP. (**B**) The results of qRT-PCR of DELs of 120 DP vs. 90 DP. The goat beta-actin (ACTB) gene was used as reference gene. Data are shown as means ± *SE* (*n* = 3). *: *p* < 0.05, **: *p* < 0.01, and ***: *p* < 0.001. (**C**) Correlation of RNA-Seq and qRT-PCR. Comparison of changes in lincRNA expression (represented as log2) derived from RNA-Seq and qRT-PCR revealed a significant correlation (Pearson’s *r* = 0.81 and *p* = 0.016 at the *α* level of 0.05).

To verify the accuracy of the transcriptome sequencing, the expression of eight randomly selected DELs was investigated by qRT-PCR test and the results were identical to those from transcriptome sequencing, which proved the reliability of the high-throughput RNA-Seq data (Figure 6).

## 4. Discussion

### 4.1. Characteristics of LincRNAs Identified in This Study

It is well known that lincRNA is widely involved in mammalian development [48]. However, the process of lincRNAs regulating the goat testis development still remains to be elucidated. In this study, 16,931 novel lincRNAs were identified in the goat testis. A previous study found that lincRNA has unique features compared with mRNA [49]. In the present study, the lincRNAs presented fewer exons (Figure 1B and Table S2), shorter transcripts (Figure 1C and Supplementary Table S2), and much lower transcriptional levels (Figure 1D and Supplementary Table S2) than the mRNAs.

Previous studies repeatedly revealed the low sequence conservation in lincRNAs among species [50,51,52]. Goat testicular lincRNAs (2504 nt on average) found in this study were longer than those of humans (1000 nt on average) and mice (550 nt on average) [53], contained fewer exons (2.88 exons on average) than mice (3.7 exons on average) but a similar exon number to humans (2.9 exons on average) [53]. Interestingly, compared with humans and mice, the lincRNAs found in this study had significantly higher sequence identity with the lincRNAs of cows (that also belong to the family Bovidae) (Figure 1E).

### 4.2. The Specific Expression Pattern of LincRNAs

LincRNA tends to express in a temporal-specific manner [54]. The expression patterns of testicular lincRNA and protein-coding genes varied greatly during the period of 0–180 DP in the present study. LincRNAs were clustered into the 0–90 DP and 120–180 DP stages using PCA and correlation analysis (Figure 2A,B), and the ratio between the DEL numbers in the periods of 90–120 and 60–90 DP was 20.44 (Figure 2C,D). In contrast, the protein-coding genes were clustered into the 0–60, 90, and 120–180 DP stages in our previous study [22]. The ratio between the DEL numbers in the periods of 90–120 and 60–90 DP was approximately 4-fold that of DEPGs (Figure 2C,D). Furthermore, the majority of ULs at 90–120 DP stage were non-differentially expressed in the periods of 0–30 DP, 30–60 DP, and 60–90 DP, which indicates that a large number of lincRNAs are activated for the first time in the 90–120 DP stage to perform certain functions specific to this stage.

In this study, the WGCNA approach was used to cluster lincRNAs into several modules, and the expression pattern of lincRNAs in each module showed distinct temporal-specific characteristics (Figure 3). Interestingly, although the number of lincRNA modules was greater than that of mRNA, the expression patterns of lincRNA modules were more monotonous than that of mRNA modules (Supplementary Figure S2), which implies a more strictly regulated expression and more specific function of lincRNAs. Similar phenomena were also observed in previous studies [54]. Considering the ambiguity of the concept of lincRNA [55], we believe that the complex lincRNA expression patterns found in this study may be partly due to the diversity of lincRNA functions.

### 4.3. The Role of LincRNAs in Goat Testicular Development

LincRNA plays physiological roles by regulating the expression of protein-coding genes. For instance, *linc-MD1*, a muscle-specific lincRNA, is involved in the muscle development by regulating the *MAML1* gene [56]. Thus, in the present study, the lincRNA function of each module was investigated through the GO enrichment of the coexpressed protein-coding genes.

#### 4.3.1. The Down-Regulation of LincRNAs at 90 DP

In this study, the lincRNAs in LM1 are mainly involved in organ-growth-related processes, including the regulation of organ growth, tubular tissue growth, epithelial morphogenesis, connective tissue growth, neurogenesis, muscle tissue development, angiogenesis, immunity, and DNA damage repair (Figure 4A and Supplementary Table S7).

Connective tissue is essential for the formation of testicular anatomy. The separation of dense connective tissue represented by the tunica albuginea is the basis for the formation of testicular lobules [57], and the fibroblasts and undifferentiated mesenchymal cells in the testis interstitium constitute the microenvironment that maintains the normal function of the seminiferous tubules [58]. Previous studies have found that the anatomical structure of the goat testis is basically formed during the organ growth phase of 0–90 DP [22], so the expression of lincRNAs that regulate the growth of connective tissue was reduced during the spermatogenesis phase after 90 DP in the present study (Figure 4A and Table S7).

The normal function of the testis requires the support of nerves, myoid, and vascular tissues. Sympathetic nerve damage leads to serious disorders of the endocrine system and spermatogenesis of the testis, and the imbalance of neurotransmitter levels in the testis is obviously related to the occurrence of infertility [59]. Myoid cells, located outside the basement membrane of spermatogenic cells, are important cells in the reproductive regulatory network with contractile and secretory functions [60]. The blood vessels in the testes branch and tortuously run around the seminiferous tubules to form a network of capillaries [60]. In this study, the expression of lincRNAs related to nerve, muscle-like tissue, and blood vessel growth was down-regulated after 90 days of age (Figure 4A and Supplementary Table S7), which accorded with their important functions in testicular growth.

Proper weakening of autoimmunity can avoid autoimmune diseases caused by the immunogenicity of sperm cells, maintain the immune-privileged environment in the seminiferous tubules, and facilitate the rapid progress of spermatogenesis [61]. The low expression of immune-related lincRNAs found in this study after 90 DP (Figure 4A and Supplementary Table S7) adds new evidence and content to this theory.

#### 4.3.2. The Up-Regulation of LincRNAs at 120 DP

In contrast to LM1, the lincRNA module LM2 exhibited low expression in the period of 0–120 DP and maintained high expression in the period of 120–180 DP. This part of lincRNAs is mainly involved in spermatogenesis, cilia growth, and DNA damage repair (Figure 4B and Table S8).

Our previous study found that goat testes transit to the spermatogenesis phase at 120 DP [22], and this study also found that spermatogenesis-related lincRNAs were up-regulated at 120 DP (Figure 4B). Vigorous spermatogenesis is ongoing in the testis after puberty initiation with extremely fast DNA synthesis and cell division, which usually means a higher incidence of tumors [62]. However, the testis did not exhibit an excessively high tumor incidence compared to other tissues [63], implying potential tumorigenesis-inhibiting mechanisms in the testis. Interestingly, the puberty modules (LM2) were overrepresented in GO terms related to cell division and DNA damage repair (Figure 4B), which may guarantee the rapid spermatogenesis and effective tumor resistance (Figure 4B and Table S8). Of course, these lincRNAs may simply involve in the meiotic processes, during which both genes involved in cell division and DNA repair are needed.

The present study clearly suggested that some lincRNAs may play vital roles in promoting the transition of testicular development from organ growth to spermatogenesis in goats. However, approaches should be used, such as single cell RNA sequencing, to identify which specific testicular cell type expresses the identified lincRNAs and the functions of these lincRNAs need further evaluation.

#### 4.3.3. The Divergent Regulation Patterns of LincRNA on Spermatogenesis and Organ Growth

In this study, the down-regulation of growth-related lincRNAs was nearly one month earlier than the up-regulation of spermatogenesis-related lincRNAs (Figure 3B,C and Figure 4A,B), while the down-regulation of growth-related protein-coding genes and the up-regulation of spermatogenesis-related protein-coding genes occurred at the same stage (Supplementary Figure S4). The anatomical structure of the testis includes tissues such as the epidermis, epithelium, nerves, blood vessels, and connective tissue. The growth of these tissues together constitutes the organ growth of the testis. In this process, numerous genes and pathways cooperate to ensure the normal growth of the testis. The smooth progress of spermatogenesis requires the coordination of many regulatory mechanisms, such as RNA methylation [64], miRNA [65], and piRNA [66]. Based on the results of this study, the down-regulated expression of protein-coding genes related to testicular growth may require the cumulative effect of lincRNAs and other regulatory factors, while spermatogenesis requires the immediate regulation of lincRNAs.

### 4.4. Prediction of the Target Genes of LincRNAs

At present, partly due to the diversity of lncRNA, many controversies still exist about the target gene prediction method of lncRNAs. Some lncRNAs exhibit an enhancer-like effect on their neighboring protein-coding genes [67]. *LincRNA-Cox2* increases the expression of the neighboring gene prostaglandin-endoperoxide synthase (*Ptgs2*) through an enhancer RNA mechanism, contributing to prostaglandin biosynthesis [68]. Most studies use 10 kb or 100 kb upstream and downstream as the estimated range of lincRNA cis-target genes [69,70]. In addition, some lncRNAs bind with mRNAs to form dimers, thereby reducing mRNA expression [67]. Therefore, mRNA that has a negative correlation with the expression of lncRNA and a low free energy of sequence binding can be regarded as a trans-target gene [67].

In the present study, regression analysis was implemented on the expression levels of lincRNAs and mRNAs to further explore the possible regulatory relationship between lincRNA and mRNAs. Massive highly positively or negatively associated genes were detected (Figure 5 and Table S10). Compared with the correlation analysis used in previous studies, the regression analysis method used in this study is more reliable for the inference of causality. Undoubtedly, the potential lincRNA–mRNA regulatory relationships also require more in-depth analysis and experimental verification.

## 5. Conclusions

Our study demonstrated the expression patterns and roles of lincRNAs during the testicular postnatal development. A dramatic phenomenon was observed that the down-regulation of growth-related lincRNAs was nearly one month earlier than the up-regulation of spermatogenesis-related lincRNAs, which is remarkably different from the expression of protein-coding genes. Additionally, potential lincRNA target genes were predicted. Moreover, key lincRNAs in the process of testicular growth and spermatogenesis were revealed by constructing the co-expression network of lincRNAs, such as ENSCHIT00000000777, ENSCHIT00000002069, and ENSCHIT00000005076. In the present study, RNA-Seq implied the divergent regulation patterns of lincRNAs on spermatogenesis and testis growth, thereby providing a further understanding of the functions of lincRNAs in testis development.

## Figures and Tables

**Table 1:** Primer sequences for qRT-PCR test.

Gene	Forward Primer	Reverse Primer
*ACTB* *	GTCACCAACTGGGACGACAT	CATCTTCTCACGGTTGGCCT
TCONS_00003665	GCTTTGAGAAACCCTGGGGAA	CTCCCTTTCCACAAGCCAAA
TCONS_00068785	GTCTCCCTGCACTGCTGTAT	ATGGGACTGCTGTCGATTCA
TCONS_00168995	AGGCCTGCCTTGTAGAGGAT	TCGTCACATGGAGAAGCAGA
TCONS_00276977	CGCTTCCTGAGGGTTTAGCA	CACACTGCCTGGTACATGGT
TCONS_00301980	AATAGGGGGAAAGCCGGTTG	CGGAGGCCATTTGACTCCTT
TCONS_00307506	CATTCTCTGAGACATTTTATC TCCC	TCAAGGTTGTATTTCTGATTG TGTG
TCONS_00349640	ATGATGTCAGTTGAGACTCA GAA	TGTGTTGCTGTTGACTTGCAG
TCONS_00360520	GAGCAGTTCTCCTGGGTTCC	GGGTCCCTTCATGATTGCCA
TCONS_00369376	AAGCTCTGTAGCCAGTGTTCC	TTTAGCCATGCTCTGAGGTCC
TCONS_00377658	ACAGCCCTGAGCGAGAAGA	AGCCTGTCCAGACGAAAGGA

* Gene *ACTB* was used as the reference gene.

## Data Availability

The raw data of transcriptome sequence have been deposited in the Genome Sequence Archive [23] in BIG Data Center [24], Beijing Institute of Genomics (BIG), Chinese Academy of Sciences (accession code: CRA002191) at https://bigd.big.ac.cn/gsa, accessed on 20 December 2019.

## Supplementary Materials

The following are available online at https://www.mdpi.com/2076-2615/11/3/625/s1, Figure S1: Hierarchical clustering heatmap of all lincRNAs by samples. The color of the heatmap represents log_2_(FPKM+1). The color of the blocks below the sample names represents the age group to which each of the samples belongs to. Figure S2: The discrete expression modules based on differentially expressed mRNAs by the WGCNA analysis. (A) Hierarchical cluster dendrogram of all mRNAs modules. Modules corresponding to branches are labeled with colors indicated by the color bands underneath the tree. (B,C) Eigengene bar plot of mRNA modules. Samples were sorted by days postnatal in the order of 0, 30, 60, 90, 120, 150, and 180 DP. Figure S3: Module-trait correlations of lincRNA and mRNA modules. Figure S4: Bubble chart presentation of the GO terms for mRNA modules. (A) Bubble chart presentation of the GO terms for mRNA in module MM1. (B) Bubble chart presentation of the GO terms for mRNA in module MM2. Color degree of each dot represents the statistical significance of pathways (−log_10_*FDR*). The size of the dot represents the number of mRNAs. Figure S5: Circular presentation of negative association between lincRNA and mRNA modules. The length of the inside cambered bar represents the number of lincRNAs and mRNAs. The outer cambered bar represents the proportion of lincRNAs and mRNAs with potential regulatory relationships to the number of total lincRNAs (LM modules) and mRNAs (MM modules) in their belonging module.Table S1: Information of the novel lincRNAs identified in this study. Table S2: The statistics of the exon number, transcript length and expression level of lincRNAs and mRNAs. Table S3: FPKM matrix of lincRNAs. Table S4: Details of DELs. Table S5: List of lincRNAs in each lincRNA module. Table S6: List of mRNAs in each mRNA module. Table S7: GO enrichment of lincRNAs in LM1. Table S8: GO enrichment of lincRNAs in LM2. Table S9: Details of hub lincRNAs in LM1 and LM2. Table S10: Regression analysis results of lincRNAs and mRNAs.
