# Supplementary figures and images for: RNA-Seq Implies Divergent Regulation Patterns of LincRNA on Spermatogenesis and Testis Growth in Goats
Source: Animals (Basel). 2021 Feb 26;11(3):625. doi: 10.3390/ani11030625 (PMC7996862; doi:10.3390/ani11030625)

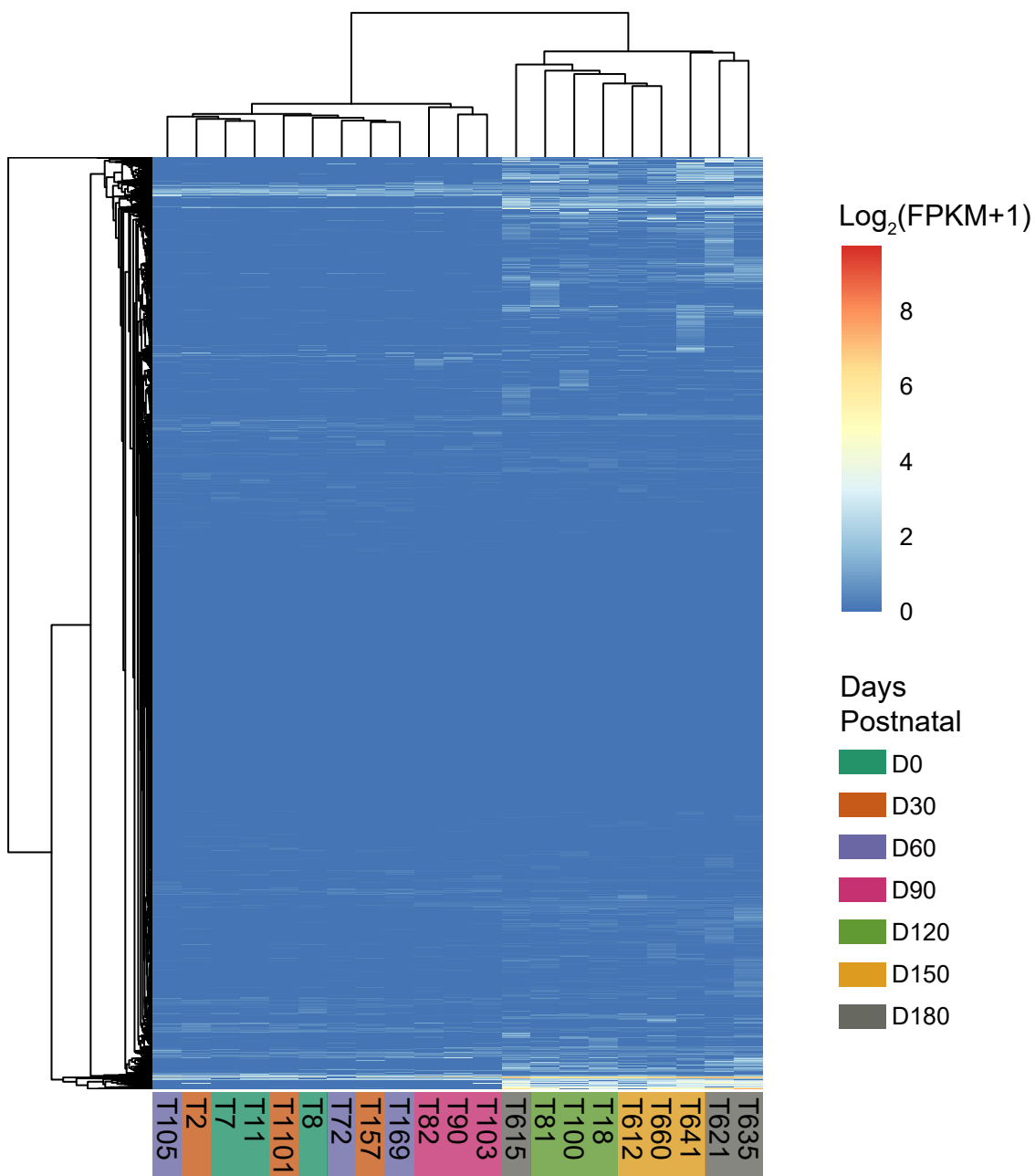

Supplement: Supplementary file 1 [file animals-11-00625-s001.zip › Figure S1.pdf]

**A**

Cluster Dendrogram

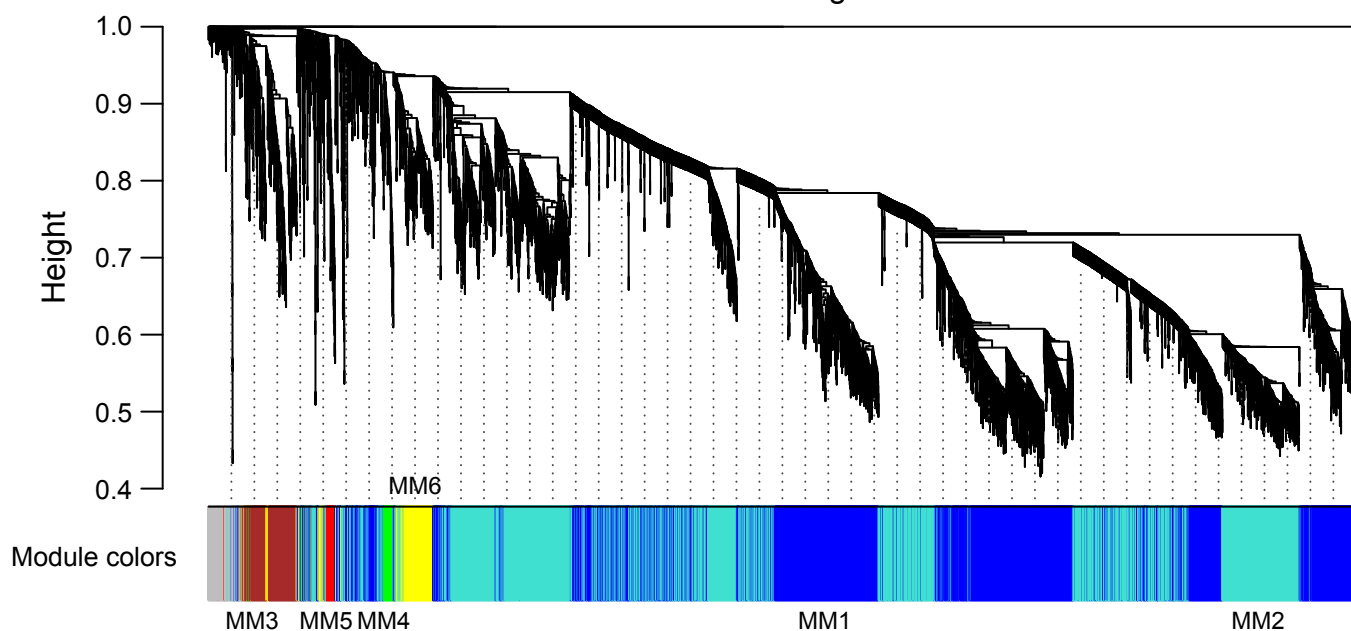**B**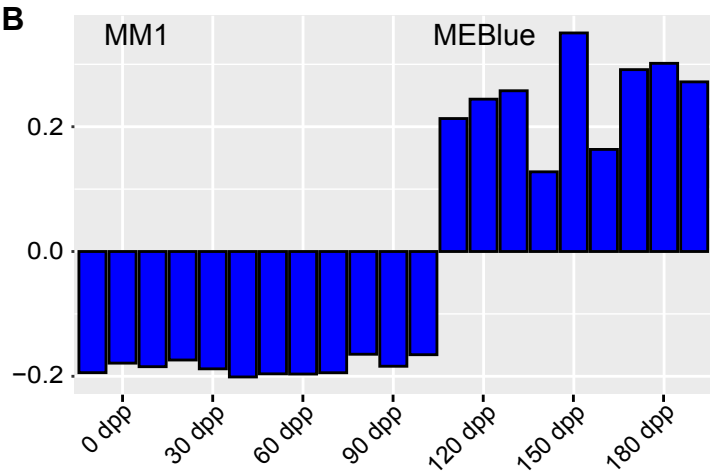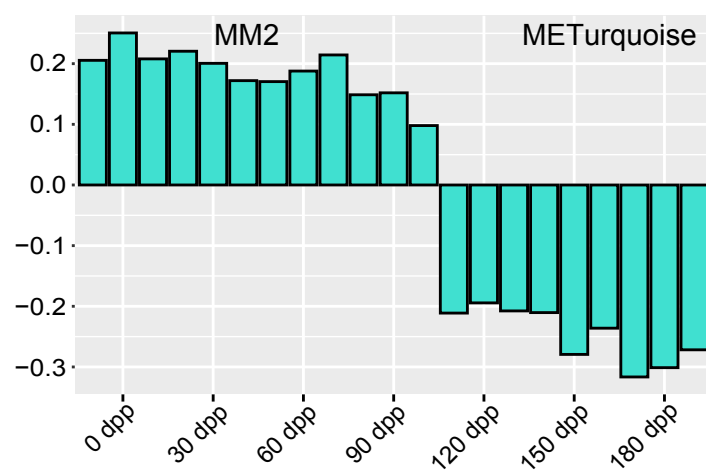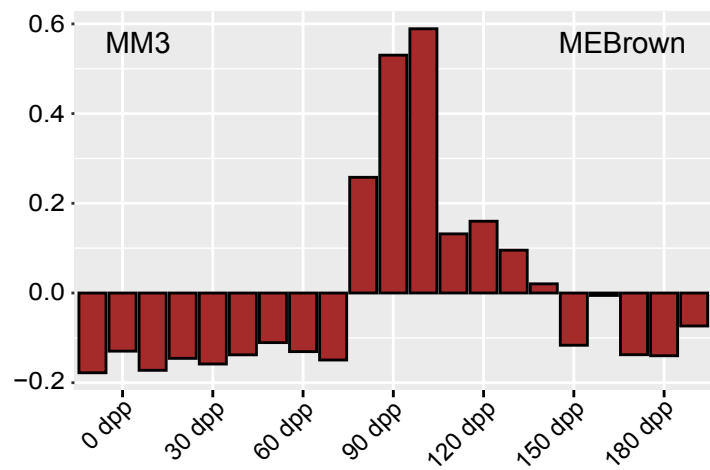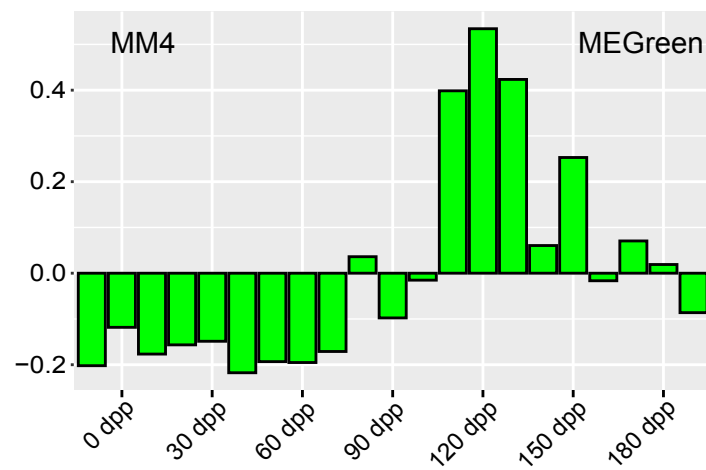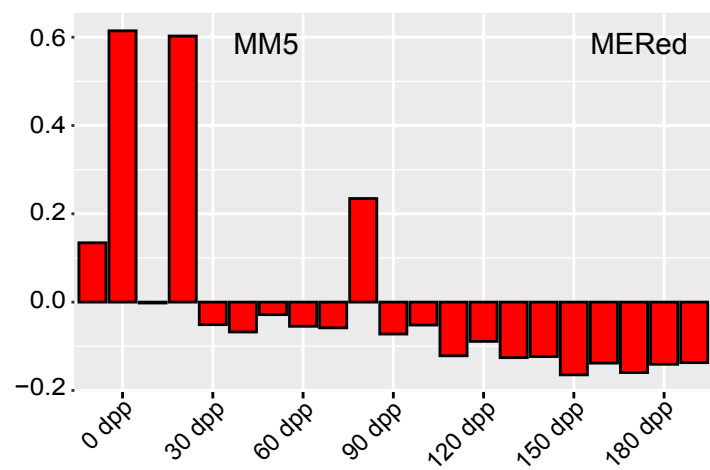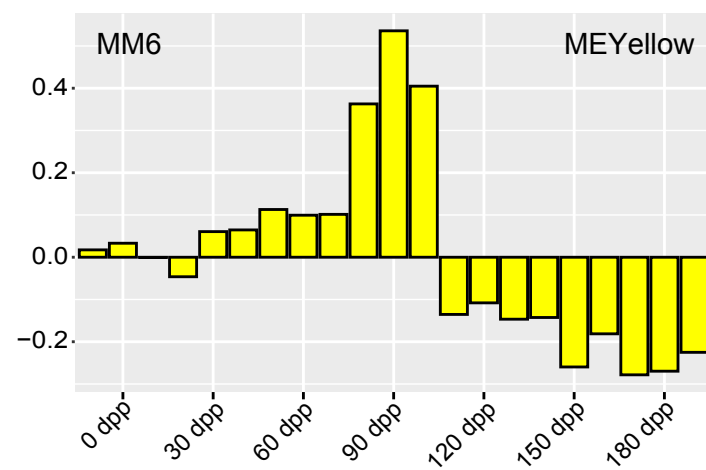

Supplement: Supplementary file 1 [file animals-11-00625-s001.zip › Figure S2.pdf]

**A**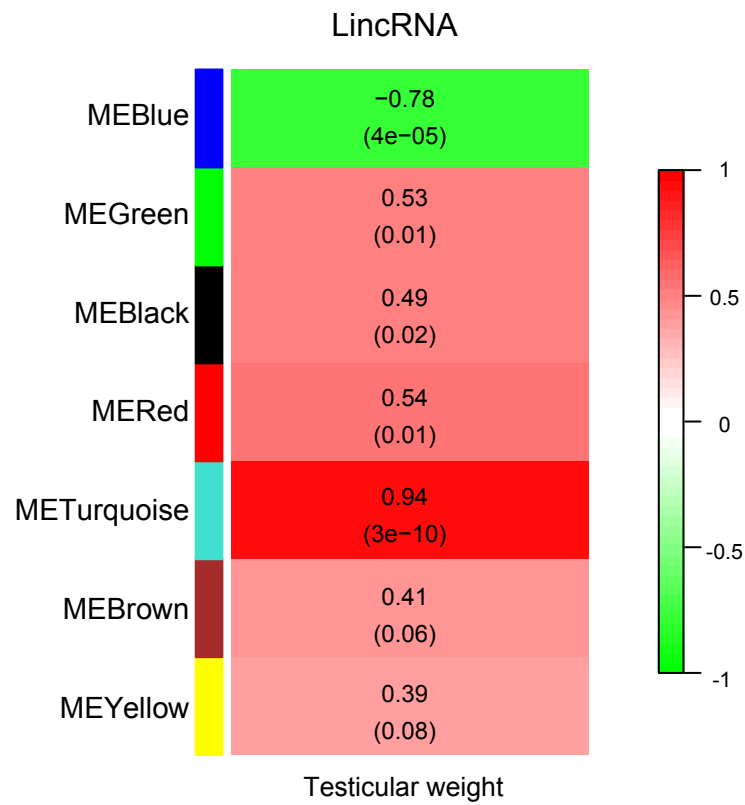**B**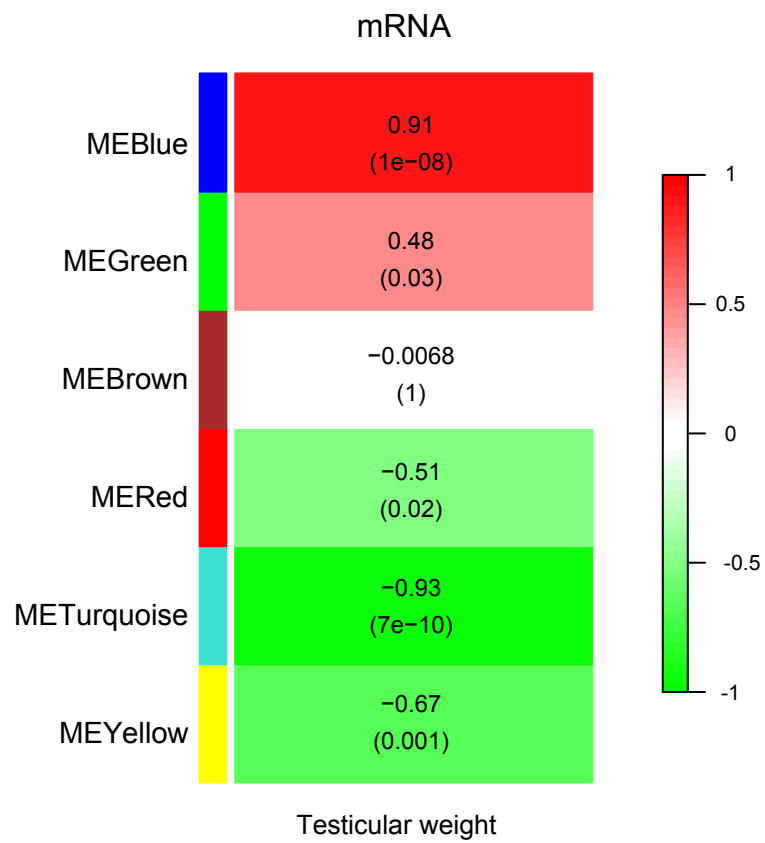

Supplement: Supplementary file 1 [file animals-11-00625-s001.zip › Figure S3.pdf]

**A**

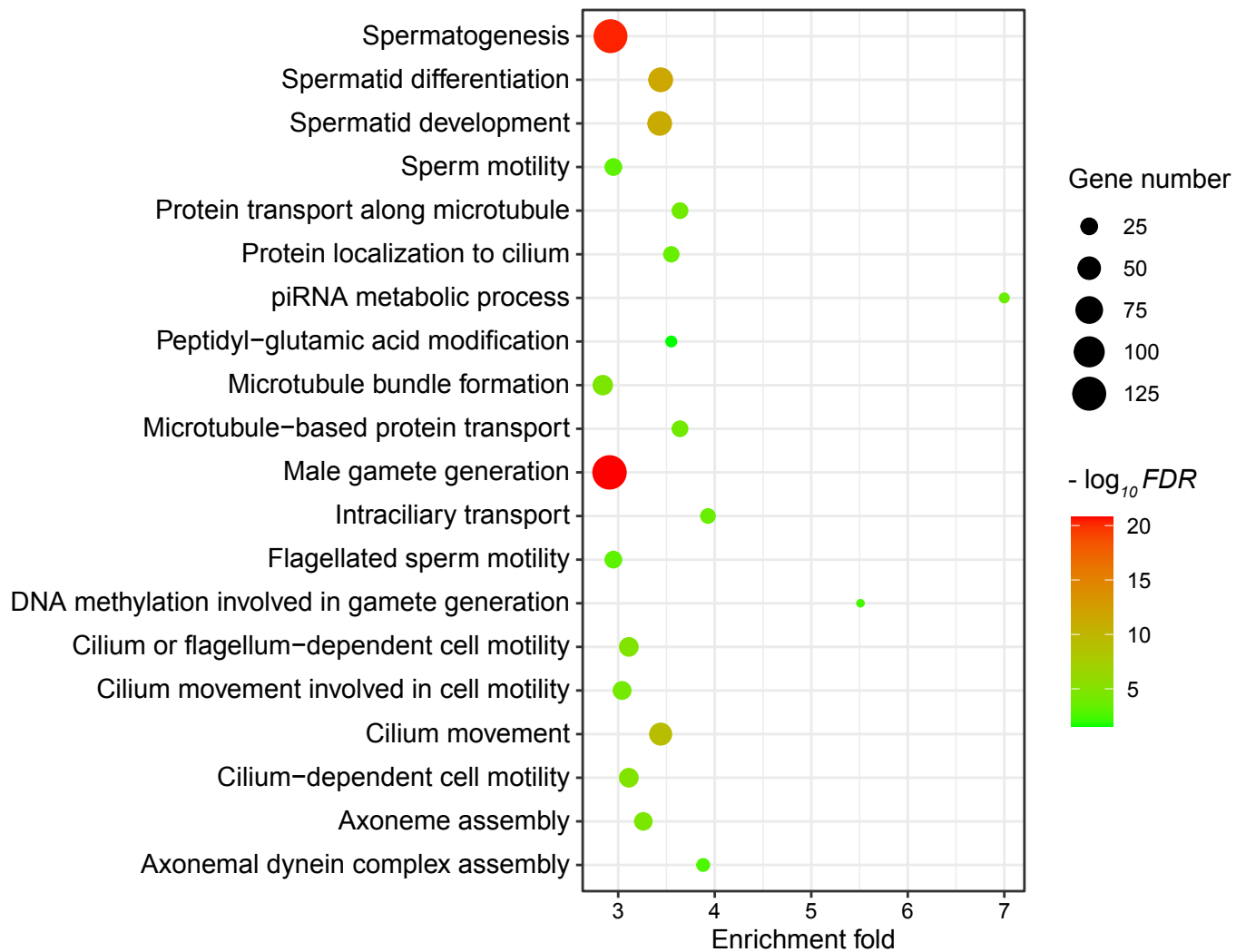

**B**

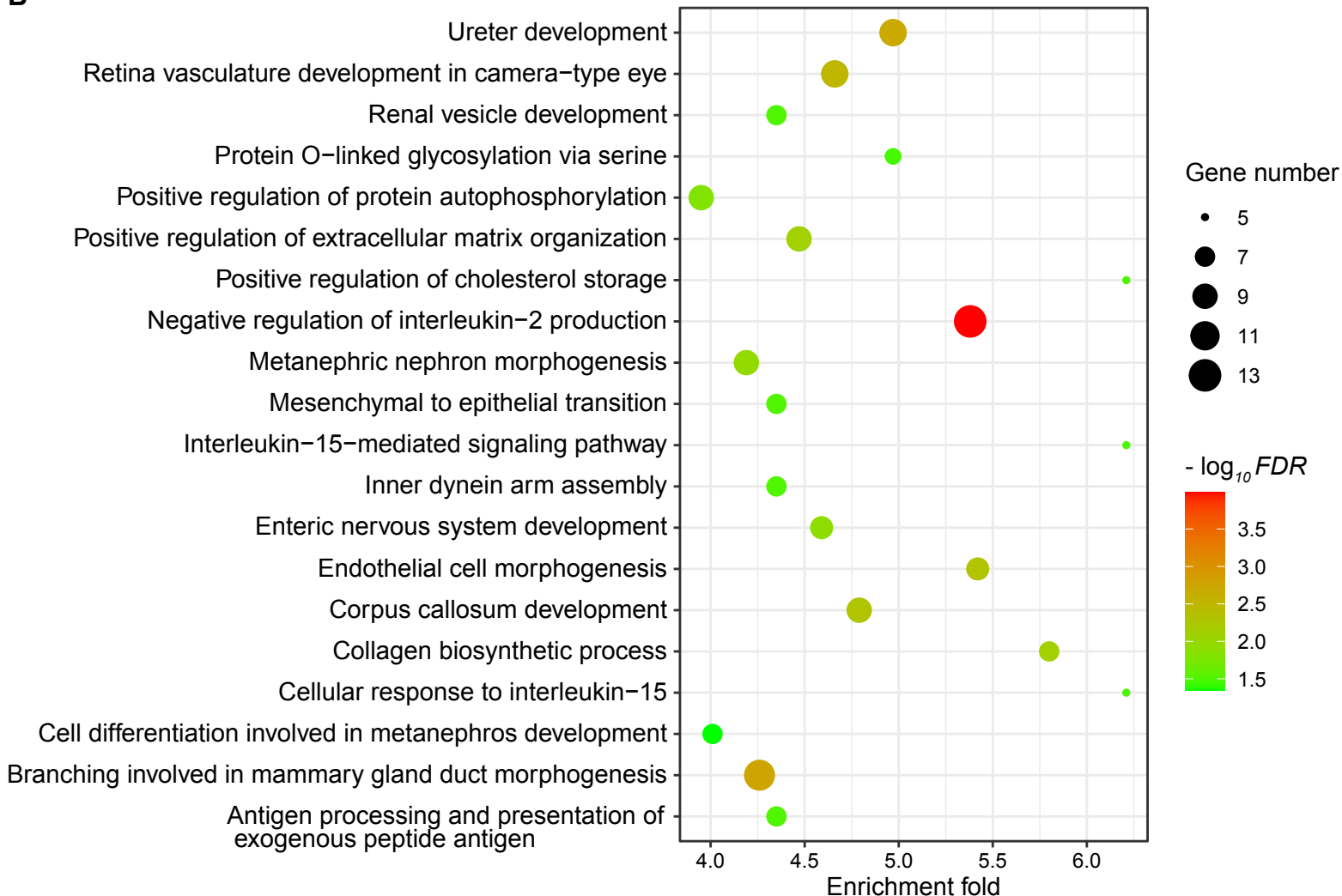

Supplement: Supplementary file 1 [file animals-11-00625-s001.zip › Figure S4.pdf]
